# A Vaccine Strain of the A/ASIA/Sea-97 Lineage of Foot-and-Mouth Disease Virus with a Single Amino Acid Substitution in the P1 Region That Is Adapted to Suspension Culture Provides High Immunogenicity

**DOI:** 10.3390/vaccines9040308

**Published:** 2021-03-24

**Authors:** Ji-Hyeon Hwang, Gyeongmin Lee, Aro Kim, Jong-Hyeon Park, Min Ja Lee, Byounghan Kim, Su-Mi Kim

**Affiliations:** Center for Foot-and-Mouth Disease Vaccine Research, Animal and Plant Quarantine Agency, 177 Hyeoksin 8-ro, Gimcheon City 39660, Korea; jihyeonh87@korea.kr (J.-H.H.); lgm6004@korea.kr (G.L.); arokim728@korea.kr (A.K.); parkjhvet@korea.kr (J.-H.P.); herb12@korea.kr (M.J.L.); kimbh61@korea.kr (B.K.)

**Keywords:** foot-and-mouth disease virus, vaccine, A/ASIA/Sea-97, immunogenicity

## Abstract

There are seven viral serotypes of foot-and-mouth disease virus (FMDV): A, O, C, Asia 1, and Southern African Territories 1, 2, and 3 (SAT 1–3). Unlike serotype O FMDV vaccine strains, vaccine strains of serotype A FMDV do not provide broad-range cross-reactivity in serological matching tests with field isolates. Therefore, the topotype/lineage vaccine strain circulating in many countries and a highly immunogenic strain might be advantageous to control serotype A FMDV. We developed a new vaccine strain, A/SKR/Yeoncheon/2017 (A-1), which belongs to the A/ASIA/Sea-97 lineage that frequently occurs in Asian countries. Using virus plaque purification, we selected a vaccine virus with high antigen productivity and the lowest numbers of P1 mutations among cell-adapted virus populations. The A/SKR/Yeoncheon/2017 (A-1) vaccine strain has a single amino acid mutation, VP2 E82K, in the P1 region, and it is perfectly adapted to suspension culture. The A/SKR/Yeoncheon/2017 (A-1) experimental vaccine conferred high immunogenicity in pigs. The vaccine strain was serologically matched with various field isolates in two-dimensional virus neutralization tests using bovine serum. Vaccinated mice were protected against an A/MAY/97 virus that was serologically mismatched with the vaccine strain. Thus, A/SKR/Yeoncheon/2017 (A-1) might be a promising vaccine candidate for protection against the emerging FMDV serotype A in Asia.

## 1. Introduction

Foot-and-mouth disease (FMD) is an acute contagious disease that affects cloven-hoofed animals such as cows, pigs, sheep, goats, and deer. It induces fever; lameness; and vesicles on the mouth, tongue, snout, teats, and feet [1,2]. The FMD virus (FMDV) belongs to the genus *Aphthovirus* of the family *Picornaviridae* and is composed of a single-stranded, positive-sense RNA genome. The virus consists of seven serotypes: A, O, C, Asia1, and South African Territories 1, 2, and 3 (SAT 1–3).

An effective FMD vaccine or vaccine strain should be antigenically similar to causative viruses and evoke powerful immune responses [3]. The FMDV, O1 Manisa, and O PanAsia-2 vaccine strains of serotype O provide broad-spectrum protection. However, vaccine strains of serotype A FMDV do not provide broad-range cross-reactivity in serological matching tests with field isolates because unlike serotype O, serotype A FMDV is antigenically diverse [4,5,6]. Therefore, the topotype/lineage vaccine strain circulating in many countries might be advantageous to control serotype A FMDV. In addition, more immunogenic vaccines are necessary to improve the range of protection [7].

Outbreaks of serotypes A and O FMDV are frequently reported worldwide [8]. A/AISA/Sea-97 was the most prevalent topotype among serotype A viruses in sporadic or endemic outbreaks in the pool 1 region (Southeast, Central, and East Asia) between 2009 and 2020. Outbreaks of FMD caused by A/ASIA/Sea-97 have been reported in various Asian countries, including Republic of Korea, Mongolia, Laos, and Thailand between 2016 and 2020. Therefore, the A/ASIA/Sea-97 lineage could be a promising vaccine strain for pool 1 region countries. In particular, outbreaks caused by the G2 sublineage of A/ASIA/Sea-97 have been frequently reported in countries of the pool 1 region, including China (2013), Russia (2014), and Vietnam (2016). FMDV A/SKR/Yeoncheon/2017, which belongs to A/ASIA/Sea-97-G2, was isolated in Republic of Korea, and is genetically highly similar to FMDV serotype A viruses circulating in Asian countries, including Vietnam, Mongolia, and Myanmar [9]. Therefore, a A/SKR/Yeoncheon/2017 vaccine strain might be a promising vaccine candidate for use in Asian countries.

Amino acid mutations that arise in the P1 region of vaccine strains after serial passages are inevitable because the integrin receptor usage of FMDV had to change to produce FMD antigens in baby hamster kidney (BHK)-21 cell suspensions, which express low levels of integrin receptors [10]. Numerous amino acid mutations in the seven FMDV serotypes have been reported [11]. The single amino acid mutation of the 56th position at VP3 is reportedly critical for cell adaptation in serotypes O and A [12,13]. The VP1 region, which includes major antigenic sites, is the most frequently studied in terms of mutations among P1 regions. Furthermore, the antigenic and receptor-interacting sites overlap in some cases, which might lower the neutralizing antibody titer [14,15,16]. Therefore, several amino acid mutations in field isolates that account for the adaptation to growth in cell culture are a practical concern to the vaccine industry. As there is a correlation between the neutralizing antibody titer and protection against FMDV, it has been considered that the humoral immune response is important in vaccine protection against FMDV [17]. Therefore, cell-adapted FMDV with a minimum number of amino acid substitutions, minimizing possible antigenic changes, might provide a good candidate vaccine strain.

Here, we developed a novel vaccine strain using the A/SKR/Yeoncheon/2017 isolate, which belongs to the A/ASIA/Sea-97 lineage, a G2 sublineage circulating in the pool 1 region. An FMD outbreak caused by A/ASIA/Sea-97 occurred in Yeoncheon county, Republic of Korea, in 2017, and the virus was effectively adapted for culture in BHK-21 suspension cells in this study. We selected a single vaccine seed virus of A/SKR/Yeoncheon/2017 (A-1) with high growth efficiency and the lowest numbers of amino acid mutations, as well as high antigen productivity, in order to develop an effective vaccine strain of serotype A FMDV. We also tested the A/SKR/Yeoncheon/2017 vaccine for immunogenicity and early protection in pigs and for protection against heterologous FMDV in mice.

## 2. Materials and Methods

### 2.1. Cells and Viruses

Porcine kidney (LFBK) cells (Plum Island Animal Disease Center, Orient, NY, USA) [18], fetal goat tongue (ZZ-R 127) cells (Friedrich-Loeffler-Institut, Riems, Germany), and baby hamster kidney (BHK)-21 adherent cells were cultured in Dulbecco’s modified Eagle’s medium (DMEM; Thermo Fisher Scientific, Waltham, MA, USA). Chinese hamster ovary (CHO)-K1 cells (ATCC CCL-61, American Type Culture Collection, Manassas, VA, USA) were maintained in Ham F-12K medium (Thermo Fisher Scientific, Waltham, MA, USA). The media were supplemented with 10% fetal bovine serum (pH 7.4), and the cells were grown at 37 °C in a 5% CO_2_ incubator. BHK-21 suspension cells were cultured in CD-BHK-21 production medium (Lonza, Basel, Switzerland) supplemented with dextran sulfate (Sigma-Aldrich, St. Louis, MO, USA), l-glutamine (Thermo Fisher Scientific, Waltham, MA, USA), pluronic F-68 non-ionic surfactant (Thermo Fisher Scientific, Waltham, MA, USA), and HyClone Cell Boost 5 Supplement (Fisher Scientific, Hampton, NH, USA) at 37 °C in a 5% CO_2_ shaking (110 rpm) incubator. The BHK-21 suspension cells were established by the Animal and Plant Quarantine Agency (APQA) and the Korea Research Institute of Bioscience and Biotechnology in Republic of Korea.

FMDV A/SKR/Yeoncheon/2017 (GenBank accession no. KR766148), belonging to the A/ASIA/Sea-97-G2 topotype/lineage-sublineage, was isolated in Yeoncheon county, Gyeong-gi-do province, Republic of Korea, in 2017 by the APQA. FMDV A/VIT/2013 (GenBank accession no. KY322680), belonging to the A/ASIA/Sea-97 topotype/lineage; A/SKR/Pocheon/2010 (GenBank accession no. KC588943), belonging to the A/ASIA/Sea-97-G1 topotype/lineage-sublineage; A/SKR/Gimpo/2018 (GenBank accession no. MK463492), belonging to the A/ASIA/Sea-97-G2 topotype/lineage-sublineage; A22/IRQ/24/64 (GenBank accession no. AY593763), belonging to the A/ASIA topotype; and A/Malaysia/97 (A/MAY/97, GenBank accession no. KJ933864), belonging to the A/ASIA/Sea-97 topotype/lineage were used for virus neutralization tests (VNTs) or two-dimensional (2D) VNTs. A22/IRQ/24/64 and A/MAY/97 were obtained from the Pirbright Institute (OIE/FAO Reference Laboratory for FMD, Woking, UK). A/SKR/Pocheon/2010 and A/SKR/Gimpo/2018 were isolated in Republic of Korea by the APQA, and A/VIT/2013 was isolated in Republic of Korea by the APQA using field samples supplied by the National Center for Veterinary Diagnosis in Vietnam. FMDV infection and culture were conducted in a Bio-Safety Level 3 facility at the APQA.

### 2.2. Serial Passaging of FMDV and Virus Plaque Purification

The A/SKR/Yeoncheon/2017 isolate was serially passaged 5 times in LFBK cells, 4 times in adherent BHK-21 cells, and 7 times in BHK-21 suspension cells for virus culture. Virus plaques were purified to obtain a single viral clone [19]. BHK-21 adherent cells in a 6-well plate were infected with the serially passaged A/SKR/Yeoncheon/2017 virus. After adsorbing the cell-adapted virus for 1 h, the cells were overlaid with melted SeaPlaque agarose (Lonza, Basel, Switzerland) mixed with neutral red (Sigma-Aldrich) and incubated at 37 °C in 5% CO_2_ for 72 h. Three viral plaques were picked, and the purified viruses in the agar plugs were resuspended in DMEM by pipetting. After centrifugation, the supernatant was used to infect adherent BHK-21 cells, which were incubated at 37 °C in 5% CO_2_. The harvested viruses were serially passaged once in BHK-21 adherent cells and twice in BHK-21 suspension cells to increase the viral titer.

### 2.3. Infectivity Tests in CHO-K1 and BHK-21 Adherent Cells

Viral titers were measured and calculated using the Reed and Muench method at a 50% tissue culture infective dose (TCID_50_) [20]. CHO-K1 cells were used to test the dependence of FMDV on heparan sulfate (HS) as a receptor [21,22]. To measure the titer in permissive cells expressing integrin, which is the receptor of wild-type FMDV, we performed virus titrations in BHK-21 adherent cells, as described previously herein.

### 2.4. Genome Amplification, Nucleotide Sequencing, and Amino Acid Sequence Alignment

Viral RNA was extracted from 100 μL of supernatant of plaque-purified A/SKR/Yeoncheon/2017 (A-1, A-2, and A-3) virus and serially passaged once in BHK-21 adherent cells and twice in BHK-21 suspension cells, as mentioned in Section 2.2, using the MagNA Pure 96 System (Roche, Basel, Switzerland). The RNA was treated using a one-step PCR inhibitor removal kit (ZYMO Research, Irvine, CA, USA), and single-stranded cDNA was prepared by reverse transcription using an oligo-(dT)_18_ primer and SuperScript II Reverse Transcriptase (Thermo Fisher Scientific, Waltham, MA, USA). The P1 (VP4, VP2, VP3, and VP1) region was PCR-amplified using Phusion High-Fidelity DNA Polymerase (Thermo Fisher Scientific, Waltham, MA, USA) and purified using ExoSAP-IT Express PCR Product Cleanup Reagent (Thermo Fisher Scientific, Waltham, MA, USA). Nucleotide sequencing was performed at Macrogen Inc. (Seoul, Republic of Korea) using an ABI 3730xl DNA analyzer (Applied Biosystems, Foster city, CA, USA). The nucleotide sequences of A/SKR/Yeoncheon/2017 wild-type virus was supplied by the Foot-and-Mouth Disease Diagnosis Division of the APQA. The amino acid sequences of the P1 region were edited using the CLC Main Workbench (version 6.9.1, Qiagen Bioinformatics, Redwood City, CA, USA) and aligned using CLUSTAL W software (version 1.8) [23].

### 2.5. Production of Inactivated Antigen

We prepared 3 shaking flasks containing BHK-21 suspension cells (3 × 10^6^ cells/mL), and the medium was replaced with CD-BHK-21 production medium before virus infection. The cells in the flasks were infected with A/SKR/Yeoncheon/2017(A-1) that had been plaque-purified and serially passaged once in BHK-21 adherent cells and twice in BHK-21 suspension cells, as mentioned in Section 2.2, at a multiplicity of infection of 0.001, and cultured in a CO_2_ shaking incubator. Inactivated FMDV antigen for the quantification of antigen and the experimental A/SKR/Yeoncheon/2017(A-1) vaccine were obtained as reported [24]. Supernatants were collected at 0, 8, 12, and 16 h post-infection, and the virus was inactivated by binary ethylenimine treatment (final concentration, 3 mM) in a shaking incubator at 26 °C for 24 h. The inactivation step was repeated at 26 °C for 24 h in a new flask. The supernatant was neutralized by treatment with sodium thiosulfate. For antigen concentration and purification, the inactivated viral supernatant was mixed with 3% Polyethylene glycol (PEG) 6000 at 4 °C overnight and centrifuged (10,000 × *g*). The pellet was resuspended in TN (Tris, 50 mM; NaCl, 100 mM) buffer (pH 7.2). The inactivated 146S antigen (intact virion of FMDV) was quantified by sucrose density gradient centrifugation [25]. Briefly, the resuspended pellet was layered onto a 15–45% sucrose density gradient and ultracentrifuged at 110,000 × *g* at 4 °C in a SW41Ti rotor (Beckman Coulter, Brea, CA, USA) for 3 h. The 146S antigen was quantified by spectrophotometry at 259 nm. The FMD antigen, purified from a layer between 30% and 35% sucrose, was placed on formvar-coated grids and negatively stained with 1% uranyl acetate. The FMD antigen was imaged with a transmission electron microscope (Hitachi 7100, Tokyo, Japan).

### 2.6. Vaccination of Pigs and Cows for Serology Test

Nine-week-old pigs and 8-week-old cows verified as negative for FMDV-neutralizing antibody (neutralizing antibody titer < 1:4) were used with the approval of the Animal Care and Use Committee of the APQA (approval no. 2018-423), and the experiment was performed in an Animal Bio-Safety Level 3 facility at the APQA [26]. A total of 3 pigs and 5 cows were intramuscularly injected with the A/SKR/Yeoncheon/2017(A-1) experimental vaccine (2 mL/dose). The pigs were given a booster injection of the vaccine at 4 weeks post-vaccination (WPV). The vaccine was formulated with the A/SKR/Yeoncheon/2017(A-1) vaccine antigen (15 µg/dose) and Montanide ISA 206 (Seppic, Paris, France) as a water-in-oil-in-water (W/O/W) double emulsion. Blood samples of pigs were used for VNTs and those of cows were used for 2D VNTs.

### 2.7. VNTs for Homologous and Heterologous Viruses 

Blood samples were collected from pigs at 1, 2, 3, 4, 5, and 6 WPV. Sera were separated and heat-inactivated at 56 °C for 30 min. VNT was carried out according to the Manual of Diagnostic Tests and Vaccines for Terrestrial Animals [27]. FMDV A/SKR/Yeoncheon/2017, passaged 4 times in LFBK cells, was used for VNT as homologous virus, and A/VIT/2013, A/SKR/Pocheon/2010, and A22/IRQ/24/64 passaged 4 times in LFBK cells were used for VNT as heterologous viruses. A neutralization reaction was performed using serially diluted serum and 100 TCID_50_ of FMDV at 37 °C for 1 h. Moreover, the neutralized viruses were placed in the microplates, and then LFBK cells were added. The microplate was incubated at 37 °C for 48–72 h to assess the cytopathic effect. The neutralizing antibody titer was calculated as the reciprocal number of the maximum dilution of serum that neutralized the 100 TCID_50_ of FMDV.

### 2.8. Vaccine Matching by 2D VNT

Two-dimensional VNT was carried out according to the Manual of Diagnostic Tests and Vaccines for Terrestrial Animals [27]. Serum samples were obtained from 5 cows vaccinated with the A/SKR/Yeoncheon/2017(A-1) experimental vaccine at 28 days post-vaccination (DPV). The same field viruses used for VNT and the A/SKR/Yeoncheon/2017(A-1) vaccine virus were used for 2D VNT. The neutralizing antibody titer of the vaccine serum for 100 TCID_50_ of each virus was estimated by regression. The r1 value, which is the antigenic relationship between the vaccine strain and the field strain, was calculated as the neutralizing antibody titer against the field strain/neutralizing antibody titer against the vaccine virus. r1 values ≥ 0.3 were interpreted as cross-protective and r1 values < 0.3 as non-protective.

### 2.9. Vaccination of C57BL/6 Mice and Challenge with Heterologous FMDV

Seven-week-old C57BL/6 female mice supplied by Cosa-Bio Co., Ltd. (Sungnam, Republic of Korea) were used with the approval of the Animal Care and Use Committee of the APQA (approval no. 2018-423), and the experiment was performed in an Animal Bio-Safety Level 3 facility at the APQA. Mice were intramuscularly injected with 0.1 mL of phosphate-buffered saline (PBS) for the non-vaccinated group or an A/SKR/Yeoncheon/2017 (A-1) experimental vaccine (*n* = 5 per group). The vaccine was formulated with 4- or 10-fold diluted A/SKR/Yeoncheon/2017 (A-1) vaccine antigen (1, 0.25, 0.063, 0.016 µg/head or 1, 0.1 µg/head) and Montanide ISA 201 (Seppic, Paris, France) as a W/O/W type. At 10 or 21 DPV, the mice were challenged by intraperitoneal injection with 0.1 mL of mouse-adapted FMDV O/VIT/2013 at 200 LD_50_ (50% of the lethal dose) [24]. All mice were observed for 7 days post-challenge (DPC). 

### 2.10. Assessment of Early Protection in Pigs Vaccinated with the A/SKR/Yeoncheon/2017(A-1)

#### 2.10.1. Vaccination and FMDV Challenge

Nine-week-old pigs verified as negative for FMDV-neutralizing antibody (virus-neutralizing (VN) antibody titer < 1:4) were used with the approval of the Animal Care and Use Committee of the APQA (approval no. 2018-423), and the experiment was performed in an Animal Bio-Safety Level 3 facility at the APQA. Seven pigs were intramuscularly injected with PBS or the experimental vaccine formulated with the A/SKR/Yeoncheon/2017(A-1) vaccine antigen (15 µg/dose), 10% aluminum hydroxide gel (Rehydragel HPA, General Chemical, Moorestown, NJ, USA), saponin from *Quillaja* bark (Sigma-Aldrich) (0.5 μg/dose), and Montanide ISA 206 as a W/O/W type [28]. The pigs were intradermally challenged with 10^5^ TCID_50_ of FMDV A/SKR/Yeoncheon/2017 that had been passaged 2 times in pigs in the heel bulb of 1 foot at 7 DPV.

#### 2.10.2. Blood Sampling and Analysis

Blood samples were collected once every 2 days, and oral swabs were collected daily from 0 to 8 DPC using the BD Universal Viral Transport Kit (BD Biosciences, Franklin Lakes, NJ, USA). FMD viral RNA was identified from viral RNA extracted from serum samples by reverse-transcription (RT) real-time PCR. The cador Pathogen 96 QIAcube HT Kit (Qiagen, Hilden, Germany) was used to extract viral RNA, and RT real-time PCR was conducted as previously reported [29]. Clinical signs were monitored daily after the challenge and were scored using the following criteria: (a) lameness (1 point); (b) vesicles in the hoof and foot (1 or 2 points for each affected hoof and foot, except the foot intradermally challenged); and (c) vesicles on the snout, lips, or tongue (1 point for each affected area) (maximum, 10 points) [30].

### 2.11. Statistical Analysis

Unpaired *t*-tests were conducted using GraphPad Prism Software (version 5.0, GraphPad Software, La Jolla, CA, USA). A *p*-value < 0.05 was considered significant.

## 3. Results

### 3.1. Virus Adaptation to BHK-21 Suspension Cells over Serial Passages

**Figure 1:**
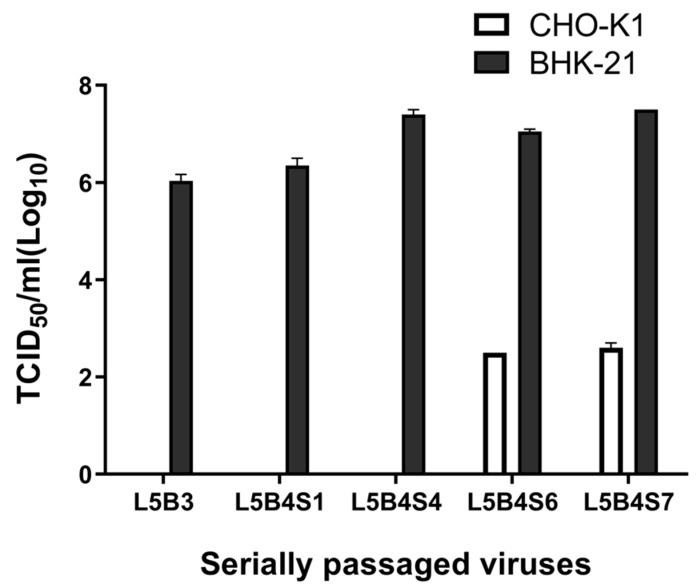
Cell adaptation of A/SKR/Yeoncheon/2017 over serial passages. Foot-and-mouth disease virus (FMDV) A/SKR/Yeoncheon/2017 was serially passaged as follows for adaptation to the cells: five times in porcine kidney (LFBK) cells and three times in baby hamster kidney (BHK)-21 adherent cells (L5B3); five times in LFBK cells, four times in BHK-21 adherent cells, and one time in BHK-21 suspension cells (L5B4S1); five times in LFBK cells, four times in BHK-21 adherent cells, and four times in BHK-21 suspension cells (L5B4S4); five times in LFBK cells, four times in BHK-21 adherent cells, and six times in BHK-21 suspension cells (L5B4S6); five times in LFBK cells, four times in BHK-21 adherent cells, and seven times in BHK-21 suspension cells (L5B4S7). The 50% tissue culture infective dose (TCID_50_) of the virus during passaging was measured in BHK-21 adherent cells expressing the integrin receptor and in CHO-K1 cells expressing the heparan sulfate (HS) receptor. Error bars indicate standard deviations (SDs) from the mean.

After five serial passages in LFBK cells, four in BHK-21 adherent cells, and seven in BHK-21 suspension cells, the viral titer of A/SKR/Yeoncheon/2017 in BHK-21 adherent cells gradually increased and reached 10^7^ TCID_50_/mL (Figure 1). A/SKR/Yeoncheon/2017 had a titer of 10^2.5^ TCID_50_/mL in CHO-K1 cells after being serially passaged five times in LFBK cells, four times in BHK-21 adherent cells, and six times in BHK-21 suspension cells (L5B4S6). These results showed that the virus became cell-adapted and employed HS as a receptor.

### 3.2. Comparison of Receptor Usage and Amino Acid Substitutions in the P1 Region in Plaque-Purified Viruses

Plaque-purified A/SKR/Yeoncheon/2017 viruses A-1, A-2, and A-3 were tested for viral titers in BHK-21 and CHO-K1 cells and for amino acid substitutions in the P1 region. The A-1, A-2, and A-3 viruses used HS as a receptor. Their titers were higher than 10^7^ TCID_50_/mL, and the titer of A-1 was the highest in BHK-21 adherent cells (Table 1). A-1, A-2, and A-3 had different amino acids substitutions in the P1 region. A-1 had a single glutamic acid-to-lysine substitution in the VP2 region, VP2 E82K. A-2 additionally had two amino acid substitutions, including a glutamic acid-to-glycine transition in the VP2 region (VP2 E131G) and VP1 K42T. A-3 had two amino acid substitutions, including a glutamic acid-to-lysine transition in the VP2 region (VP2 E131K).

### 3.3. Qualification of A/SKR/Yeoncheon/2017 (A-1) Virus for Vaccine Production 

**Figure 2:**
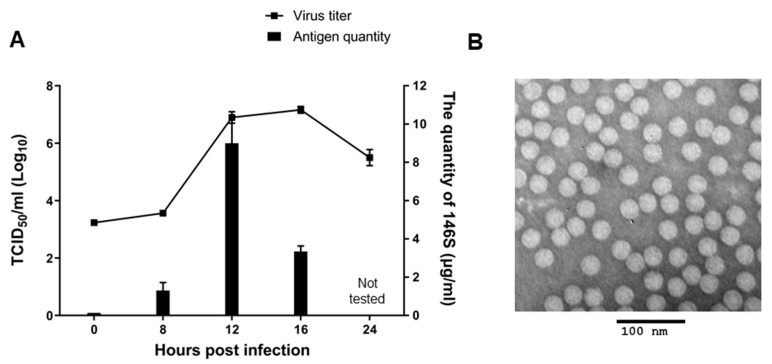
Growth of cell-adapted A/SKR/Yeoncheon/2017 (A-1) and the production of 146S antigen in BHK-21 suspension cells. (**A**) BHK-21 suspension cells were infected with cell-adapted A/SKR/Yeoncheon/2017 (A-1) at a multiplicity of infection of 0.001 and cultured at 37 °C in 5% CO_2_. Supernatants were collected at 0, 8, 12, 16, and 24 h post-infection (hpi) and the TCID_50_ was determined (line, left *Y*-axis). Inactivated antigen (146S) was quantified in the supernatants collected at 8, 12, and 16 hpi by sucrose density gradient centrifugation (bar, right *Y*-axis). Error bars indicate SDs from the mean. (**B**) Inactivated antigen (146S) from the supernatant collected at 16 hpi was confirmed by transmission electron microscopy.

Viral growth kinetics and inactivated antigen (146S) production in BHK-21 suspension cells after infection with A/SKR/Yeoncheon/2017 (A-1) were assessed for the qualification of A/SKR/Yeoncheon/2017 (A-1) virus as a vaccine seed virus (Figure 2). The A/SKR/Yeoncheon/2017 (A-1) virus titer was 10^7^ TCID_50_/mL at 12 h post infection (hpi) and was maintained until 16 hpi in BHK-21 suspension cells (Figure 2A). The highest quantity of inactivated antigen (146S) was produced at 12 hpi in BHK-21 suspension cells, and it was approximately 8 μg/mL of culture supernatant. Intact 146S antigen was observed by transmission electron microscopic imaging (Figure 2B).

### 3.4. Neutralizing Antibody Response after Immunization with A/SKR/Yeoncheon/2017 Experimental Vaccine for Homologous and Heterologous Viruses in Pigs

**Figure 3:**
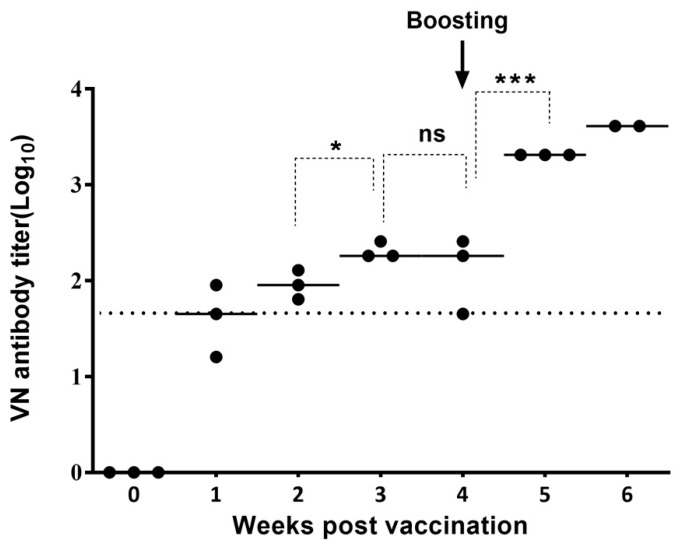
Induction of virus-neutralizing (VN) antibody in pigs vaccinated with the A/SKR/Yeoncheon/2017 (A-1) experimental vaccine. The virus neutralization test (VNT) was carried out using sera collected from pigs vaccinated with A/SKR/Yeoncheon/2017 (A-1) at 0, 1, 2, 3, 4, 5, and 6 weeks post-vaccination. The dotted line indicates the 1:45 (log_10_ 1.65) VN antibody titer cut-off level. Statistical analysis was conducted using an unpaired *t*-test (* *p* < 0.05, *** *p* < 0.005, ns: not significant). “Boosting” indicates the second vaccination.

**Figure 4:**
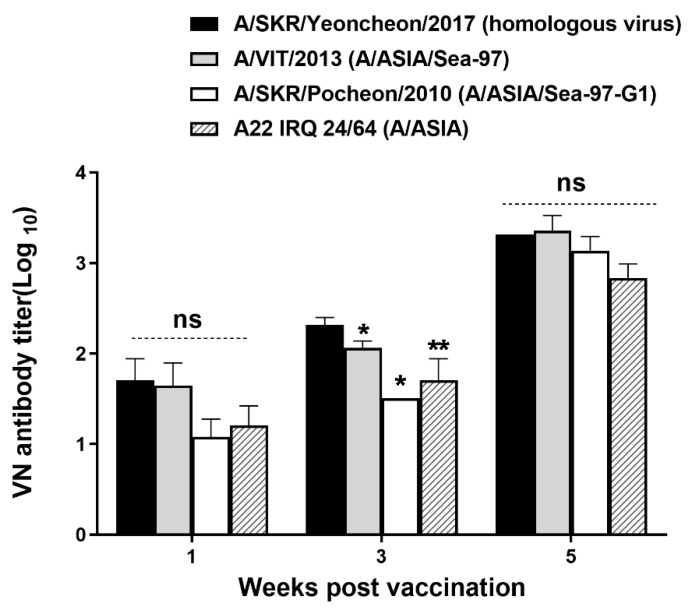
Comparisons of neutralizing titers against heterologous field viruses in pigs. The virus neutralization test (VNT) was carried out using sera from pigs vaccinated with the A/SKR/Yeoncheon/2017 (A-1) experimental vaccine and A/SKR/Yeoncheon/2017(homologous virus), A/VIT/2013, A/SKR/Pocheon/2010, and A22/IRQ/24/64, and VN antibody titers were compared. Error bars indicate SDs from the mean. Statistical analysis was conducted using an unpaired *t*-test (* *p* < 0.05, ** *p* < 0.01, ns: not significant).

To analyze the neutralizing antibody response after vaccinating pigs with the A/SKR/Yeoncheon/2017 (A-1) experimental vaccine formulated with the ISA 206 adjuvant VNT was performed for the A/SKR/Yeoncheon/2017 field virus using pig sera collected from 0 to 6 WPV (Figure 3). A VN antibody titer of ≥ 1:45 was observed in all pig sera (3/3) at 2 WPV, and a VN antibody titer of ≥ 1:100 was observed in all pig sera (3/3) at 3 WPV for homologous virus. The VN titer increased until 3 WPV and remarkably increased after boosting (VN antibody titer ≥ 1:1000; *p* < 0.05, *t*-test). In addition, VNT for heterologous viruses included in the A/ASIA topotype was performed on the serum samples collected at 1, 3, and 5 WPV (Figure 4). The VN antibody titer for heterologous field viruses was not significantly different form the VN antibody titer for homologous field virus (*p* > 0.05, *t*-test) at 1 and 5 WPV. However, The VN antibody titers for the heterologous field viruses were different from that for homologous field virus (*p* < 0.05, *t*-test) at 3 WPV. VN antibody titers for A/VIT/2013 and A/SKR/Pocheon/2010 were > 1:1000 and that for A22/IRQ/24/64 was > 1:500 in all pig sera after boosting (5 WPV).

### 3.5. Vaccine Matching Using Vaccinated Cow Sera

**Figure 5:**
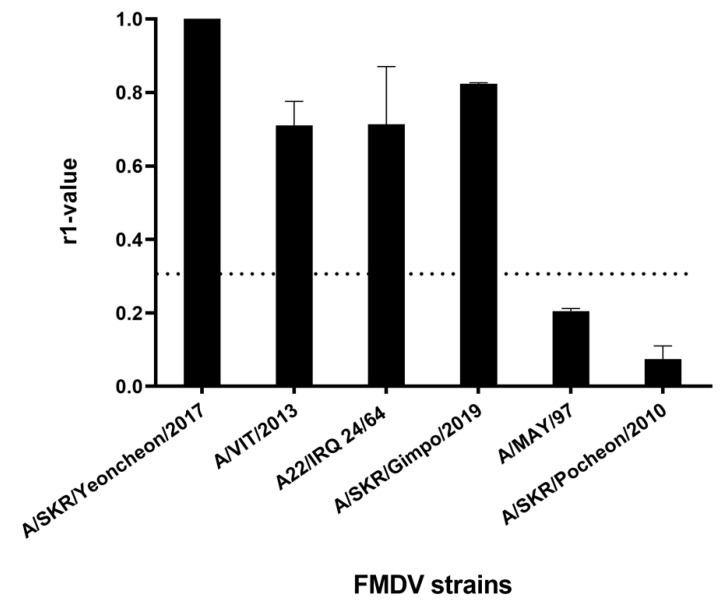
Serological cross-reactivity against heterologous viruses. A two-dimensional (2D) virus neutralization test (VNT) was performed using sera from cows vaccinated with the A/SKR/Yeoncheon/2017 (A-1) experimental vaccine and field viruses including A/SKR/Yeoncheon/2017 (A/ASIA/Sea-97-G2, homologous field virus), A/VIT/2013 (A/ASIA/Sea-97), A22/IRQ/24/64 (A/ASIA), A/SKR/Gimpo/2017 (A/ASIA/Sea-97), A/MAY/97 (A/ASIA/Sea-97), and A/SKR/Pocheon/2010 (A/ASIA/Sea-97-G1). The dotted line indicates the cut-off value of 0.3, above which the vaccine is considered to antigenically match with the field virus. Error bars indicate SDs from the mean. Experiments were performed twice independently.

Cross-reactivity with heterologous field viruses included in the ASIA topotype was assessed via 2D VNT using sera from five cows inoculated with the A/SKR/Yeoncheon/2017 (A-1) experimental vaccine formulated with the ISA 206 adjuvant at 4 WPV. The r1 value between the A/SKR/Yeoncheon/2017 vaccine strain (A-1) and the heterologous viruses A/VIT/2013, A22/IRQ/24/64, and A/SKR/Gimpo/2018 was > 0.3 (Figure 5). However, that between A/SKR/Yeoncheon/2017 vaccine strain and A/MAY/97 and A/SKR/Pocheon/2010 was < 0.3, although these viruses are included in the ASIA/Sea-97 topotype/lineage. In particular, the r1 value for A/SKR/Pocheon/2010, which is included in the A/ASIA/Sea-97/G1 sublineage, was the lowest.

### 3.6. Heterologous Virus Challenge in Vaccinated Mice

**Figure 6:**
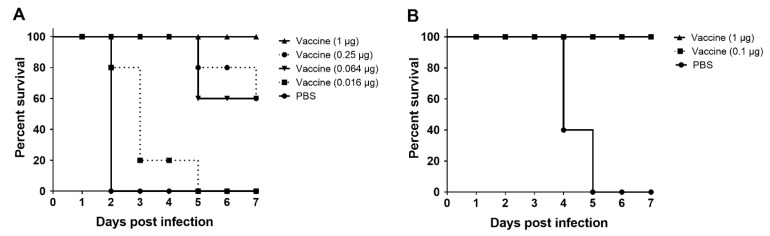
Heterologous virus challenge in mice vaccinated with the A/SKR/Yeoncheon/2017 (A-1) experimental vaccine. C57BL/6 mice were intramuscularly injected with the A/SKR/Yeoncheon/2017 experimental vaccine or phosphate-buffered saline (PBS). Each animal was inoculated with vaccine containing serially 1/4 diluted antigen (1, 0.25, 0.063, or 0.0016 μg) per animal (**A**) or 1 or 0.1 μg of antigen per animal (**B**). The animals were challenged with 200 LD_50_ of mouse-adapted FMDV A/MAY/97 via intraperitoneal injection at 10 (**A**) or 21 (**B**) days post-vaccination (DPV). Survival was monitored for 7 days.

Mice vaccinated with the A/SKR/Yeoncheon/2017 (A-1) experimental vaccine formulated with the ISA 201 adjuvant were challenged with A/MAY/97 virus, which was not antigenically matched with A/SKR/Yeoncheon/2017 (A-1) (Figure 6). In mice vaccinated with vaccine including 1 μg of antigen per head, which is the vaccine dose equivalent to 1/15 of a pig dose in the FMDV A/MAY/97 challenge at 10 DPV, the survival rate was 100% (Figure 6A). The survival rate after challenge with FMDV at 21 DPV was 100% for mice injected with vaccine containing > 0.1 μg antigen per mouse (Figure 6B).

### 3.7. Early Protection in Pigs Injected with the A/SKR/Yeoncheon/2017 (A-1) Experimental Vaccine

Pigs vaccinated with the A/SKR/Yeoncheon/2017 (A-1) experimental vaccine formulated with the ISA 206 adjuvant, aluminum hydroxide gel, and saponin were subjected to FMDV challenge with pig-adapted A/SKR/Yeoncheon/2017 virus at 7 DPV. Half of the vaccinated pigs (2/4) were protected at 7 DPV, and two pigs showed a few clinical signs (Table 2). Viral RNA was not detected in serum samples of the two pigs (no. 3 and no. 4) that were clinically protected, and a significantly low viral RNA level (about 10 copies) was detected in the serum of pig no. 2. The VN antibody titers in pigs no. 2, no. 3, and no. 4 were > 1:180 at 7 DPV. In pig no. 1, the serum viral RNA level was lower than that in the PBS control group. However, the viral RNA level in pig no. 1 was 100 times that in pig no. 2, and the VN antibody titer was only 1:16. The three pigs in the PBS control group showed severe clinical signs and high serum viral RNA levels.

## 4. Discussion

The most frequent lineage among serotype A FMDV in the pool 1 region is A/ASIA/Sea-97, accounting for about 25% of the total number of animals with FMD infected with all serotypes between 2019 and 2020 [31]. A/SKR/Yeoncheon/2017 belongs to the A/ASIA/Sea-97 lineage and is similar to strains occurring in various Asian countries, such as Vietnam, Myanmar, Thailand, and Russia [9]. However, several strains of the A/ASIA/Sea-97 lineage occurring in Southeast Asia do not match the internationally applied FMD vaccine strains such as A22 Iraq, A MAY 97, A TUR 06, and A IRN 05 in 2D-VNT by the FMD World Reference Laboratory [32]. Therefore, A/SKR/Yeoncheon/2017 might be useful as a vaccine strain for use in Asian countries.

Amino acid substitutions in the capsid of FMDV serotype A have been observed in various regions, such as the HS-binding pocket, fivefold symmetry axis, and G-H loop, which can harbor antigenically significant residues [11,13,33,34]. The most frequently reported amino acid mutation in serotype A FMDV is VP2 E131K [10]. Residues 130 and 131 are part of the EF loop and are important antigenic sites [14]. Furthermore, amino acid changes in the VP2 region are frequently reported to occur in combination with mutations in VP1 or VP3 [35,36,37]. In contrast, an amino acid substitution at position 82 of VP2 has been reported in only a few studies on serotype A FMDV [13,34].

We showed that a A/SKR/Yeoncheon/2017 (A-1) vaccine strain harboring only the VP2 E82K substitution in the P1 region uses HS as a receptor in BHK-21 suspension cells and that its 146S antigen production was suitable for large-scale production as the purified 146S antigen was > 2 μg/mL of culture supernatant. Therefore, we suggest that the amino acid substitution at position 82 of VP2 might be critical for adaptation in BHK-21 suspension cells, although it has not been frequently reported. The change of the negatively charged E to the positively charged K amino acid might be advantageous for binding to the HS receptor, as an increased number of surface positive charges has been correlated with increased interaction with the negatively charged HS receptor [14,38]. Residue 82 of VP2 is located in the surface-exposed VP2 βC strand and has been detected in serotype A virus serially passaged in BHK-21 cells [13]. In that study, VP2 E82K was accompanied by VP1 Q157R in cell-adapted serotype A FMDV. In cell-adapted A22 IRQ virus, it has been speculated that E82G on the surface of VP2 might change the structure of the VP1 and VP3 GH loops [34]. Therefore, a substitution at VP2 82 might affect receptor attachment and antigenicity. However, we only observed a substitution in VP2 E82K, but not any in VP1 or VP3, in the A/SKR/Yeoncheon/2017 (A-1) vaccine strain. Therefore, we suggest that the antigenic characteristics of this vaccine strain might be similar to those of wild-type virus because the critical antigenic sites, including the VP1 GH loop, are not altered. We also observed an amino acid substitution, A93T, in a non-structural region (2C) of the A/SKR/Yeoncheon/2017 (A-1) vaccine strain (data not shown). Amino acid substitutions in the 2C region have been reported in cell-adapted picornavirus [39,40,41]. The possibility that the substitution in the 2C region plays a role in optimization of the replication processes in different environments cannot be excluded, as amino acid substitutions in the 2C region have been observed to be critical for FMDV to adapt to a new host [42].

Cell adaptation of FMDV is achieved through selective pressure on the viral quasispecies and increased cell tropism due to genetic variation [43,44]. Therefore, we performed virus plaque purification after cell adaptation through serial passages, and we obtained three purified viruses with different characteristics. The three cell-adapted viruses (A-1, A-2, A-3) had different plaque phenotypes and 146S production levels (data not shown), as well as growth efficiencies. A-1 yielded the highest viral titer and 146S production. Furthermore, it had the lowest number of amino acid substitutions in the P1 region. A-2 and A-3 had a common amino acid substitution, VP2 E131G/K, and substitution at this position has been frequently reported [10,12,34,45]. We found no amino acid mutations except VP2 E82K in the P1 region after two additional passages of the A-1 vaccine seed virus in BHK-21 suspension cells using CD-BHK-21 medium (data not shown). However, the possibility that additional alterations occur when the virus is cultured in different cells or media cannot be excluded.

A highly immunogenic (high-potency) vaccine is important for protection against FMDV. FMD vaccine-induced immune system stimulation is influenced by the amount of antigen, antigenicity of the strain, adjuvant, and vaccine formulation [3]. We tested experimental vaccines, not a commercial vaccine, and various adjuvants, and the same A/SKR/Yeoncheon/2017(A-1) antigen was used in the experimental vaccine because the purpose of this study was to develop a novel vaccine strain with high immunogenicity (the antigenicity of the vaccine strain). Although we used various adjuvants, we observed high VN antibody titers and protection rates in all animals. Therefore, we will report the immunogenicity and potency of the final version of the A/SKR/Yeoncheon/2017 vaccine formulation in a further study.

For serotype A FMDV, the VN antibody titer at which animals are protected with 95% probability has been estimated to be approximately log_10_ 2.1 in cows [46]. We observed a maximum VN antibody titer of 1:1024 (log_10_ 3.3) at 21 DPV (1 μg antigen/dose) in mice (data not shown) and of 1:256 (log_10_ 2.4) at 21 DPV (15 μg antigen/dose) in pigs. In the early protection experiment in pigs, we observed no or low-level (score 2) clinical signs in pigs vaccinated and challenged with FMDV at 7 DPV. In addition, the VN antibody titer was > 1:100 in three of four pigs. Therefore, we suggest that vaccination with the A/SKR/Yeoncheon/2017 vaccine formulated with 15 μg antigen, ISA 206, 10% aluminum hydroxide gel, and saponin [28] might effectively provide early protection at 7 DPV, as well as protection after a second vaccination, in pigs. Because early protection is related not only to the vaccine strain, but also to the adjuvant used, the early protection rate might be enhanced through optimization of the vaccine formulation.

Cross protection has been recently reported in cattle vaccinated with an emergency vaccine against serotype A FMDV [47]. Cattle vaccinated with an A/MAY/97 vaccine poorly matching with an A/ASIA/Sea-97 lineage virus were protected against the A/ASIA/Sea-97 lineage virus. In line with this, we observed that mice vaccinated with the A/SKR/Yeoncheon/2017 experimental vaccine were protected against an A/MAY/97 virus not matching the vaccine strain. 

## 5. Conclusions

We used virus plaque purification to select a virus with high antigen productivity and the fewest amino acid mutations in the P1 region among cell-adapted virus populations. We thus selected a new vaccine strain, A/SKR/Yeoncheon/2017 (A-1), which belongs to the A/ASIA/Sea-97 lineage that frequently occurs in the pool 1 region. We showed that this vaccine strain harbored only the VP2 E82K substitution in the P1 region and provided high immunogenicity in pigs. The novel A/SKR/Yeoncheon/2017 (A-1) strain is a promising vaccine candidate for protection against the emerging FMDV serotype A in Asia.

## Figures and Tables

**Table 1:** Amino acid substitutions in the P1 region in plaque-purified viruses.

Virus ^a^	Virus Titer in Two Cell Types (TCID_50_/mL)		Amino Acid Substitutions in the P1 ^d^			
	BHK-21	CHO-K1 ^b^	VP2			VP1
			80	82	131	42
Wild-Type	NT ^c^	NT	E	E	E	K
Plaque-purified A-1	1.2 × 10^8^	5.0 × 10^2^	**·**	K	**·**	**·**
Plaque-purified A-2	2.0 × 10^7^	3.1 × 10^2^	**·**	K	G	T
Plaque-purified A-3	1.2 × 10^7^	3.1 × 10^2^	G	**·**	K	**·**

^a^ A-1, A-2, and A-3 viruses obtained by plaque purification in BHK-21 adherent cells were serially passaged once and twice in BHK-21 adherent cells and in BHK-21 suspension cells, respectively. ^b^ Virus titration was performed in Chinese hamster ovary (CHO)-K1 cells to test usage of the HS receptor. ^c^ NT: not tested. ^d^ Dots represent amino acids identical to those of the A/SKR/Yeoncheon/2017 wild-type virus.

**Table 2:** Early protection of pigs vaccinated with the A/SKR/Yeoncheon/2017 (A-1) experimental vaccine and challenged with homologous FMDV at 7 DPV.

Injection	Animal No.	Maximum Clinical Score (DPC of the First Detection) ^a^	Maximum Amount of Viremia ^b^ (DPC of the First Detection)	VN Antibody Titer (log10) at 0 DPC
Vaccine	1	2 (7)	2.7 ± 0.7 × 10^3^ (4)	1.2
	2	2 (5)	1.7 ± 1.0 × 10^1^ (6)	2.4
	3	Neg **^c^**	Neg	2.3
	4	Neg	Neg	2.4
PBS	5	10 (3)	1.8 ± 0.5 × 10^5^ (2)	<0.9
	6	10 (3)	2.5 ± 0.4 × 10^5^ (4)	<0.9
	7	10 (3)	4.5 ± 0.02 × 10^4^ (2)	<0.9

**^a^** Data (copy number of FMDV/mL) represent the mean ± SD from triplicate RT-qPCR measurements. **^b^** DPC: days post challenge. **^c^** Neg: no viral RNA and no clinical signs were detected.

## Data Availability

Not applicable.
